# Correction: Plant defense phenotypes determine the consequences of volatile emission for individuals and neighbors

**DOI:** 10.7554/eLife.16606

**Published:** 2016-04-25

**Authors:** Meredith C Schuman, Silke Allmann, Ian T Baldwin

Schuman MC, Allmann S, Baldwin IT. 2015. Plant defense phenotypes determine the consequences of volatile emission for individuals and neighbors. *eLife* **4**:e04490. doi: 10.7554/eLife.04490.

Published April 15, 2015

It came to our attention that the originally published Figure 11 contained a cosmetic and an omission error which may have been misleading. Specifically, on the right-hand side of panel C, the red bar in the middle should be pink (color representing lox2/3xTPS10) and the label below this bar is missing. These were oversights which occurred when the figure was being finalized (color scheme and labels applied). The size of the bar in question and the numbers shown in the bar are correct, as shown by comparison to the accompanying supplemental table. In the corrected figure, the color of this bar has been corrected and the missing label has been added.

The corrected Figure 11 is shown here:

**Figure:**
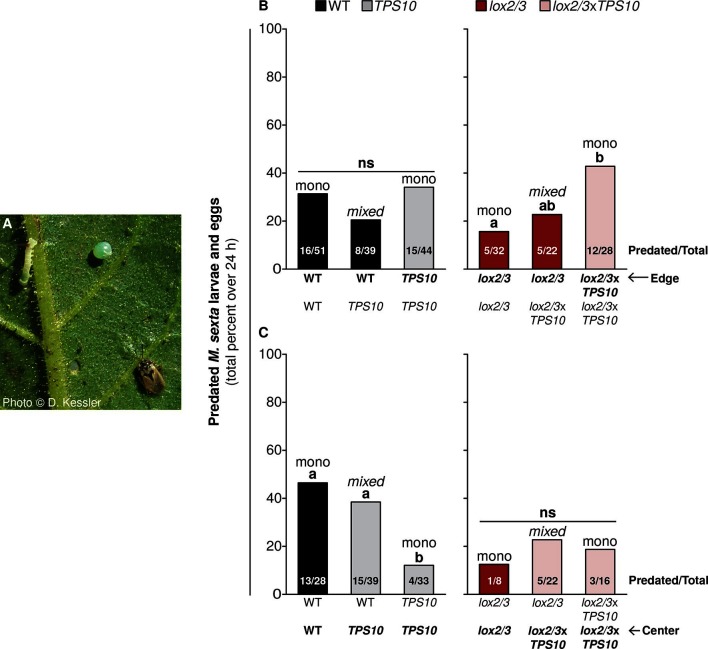

The originally published Figure 11 is shown for reference:

**Figure:**
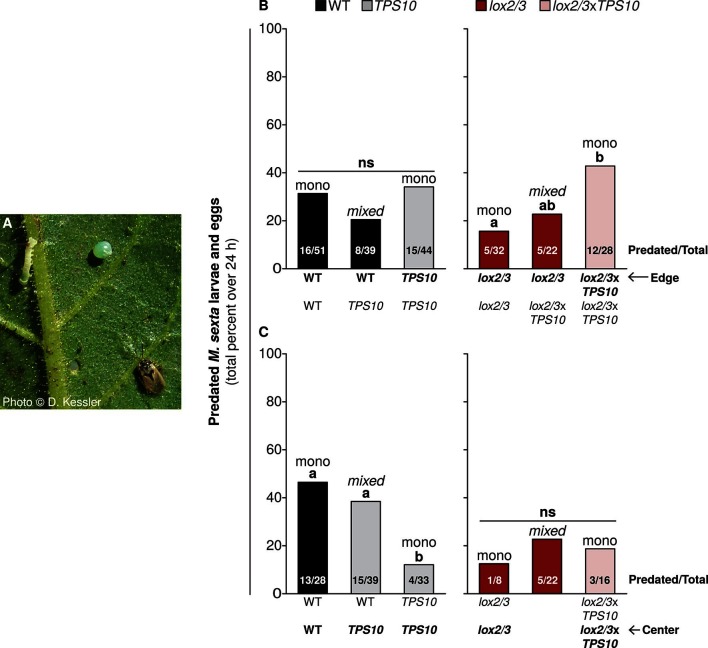

In the process of correcting this error, we thoroughly reviewed the manuscript, and we identified and corrected the following additional errors:

In Figure 2—figure supplement 1, panel B was taken at the same time as panel A and not in June. The original panel B was the alternative under consideration for Panel A. Panel B has been replaced with a photograph taken later of a similar portion of the experiment as in Panel A, as originally intended.

The corrected Figure 2—figure supplement 1 is shown here:

**Figure:**
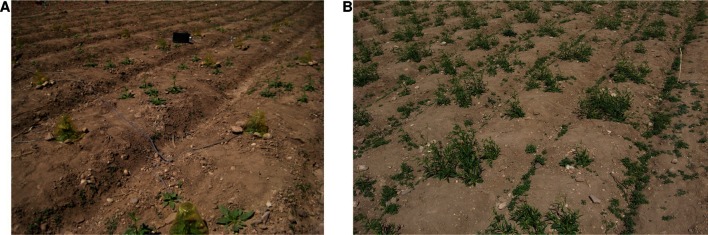

The originally published Figure 2—figure supplement 1 is shown here:

**Figure:**
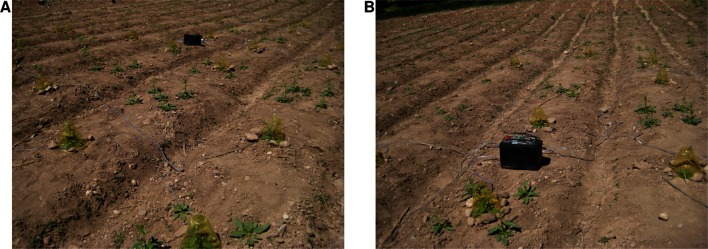

The corrected figure legend for Figure 2—figure supplement 1 is shown here:

**Photographs of the field experiment in season one**. "(**A**) Photograph of a closed-headspace volatile trapping showing ca. 25% of the experiment on May 7th of season one. (**B**) Photograph showing a similar portion of the experiment on May 23rd of season one."

The originally published figure legend for Figure 2—figure supplement 1 is shown here:

**Photographs of the field experiment in season one**. "(**A**) Photograph of a closed-headspace volatile trapping showing ca. 25% of the experiment in May of season one. (**B**) Photograph showing a similar portion of the experiment in June of season one."

In Appendix 3, company information is missing from the following sentence:

"For the volatile data in Appendix Table 1, heat- and light-resistant transparent plastic cones with open tops (frost protection cones, company) …".

This sentence has been updated to read:

"For the volatile data in Appendix Table 1, heat- and light-resistant transparent plastic cones with open tops (frost protection cones, Merox) …”.

In the Results section entitled ‘LOX2/3 deficiency accelerates flowering under low herbivory, while TPS10 expression reduces flower production under high herbivory’ a reference to Figure 8—source data 1 is missing. In an earlier version of the manuscript, the statistical analysis of this data was not mentioned until a few paragraphs later, when the remaining data set was discussed. To correct this we have now added the following sentence to the end of the first paragraph of this section:

"Statistical analyses of plant height are given in Figure 8—source data 1.”

We apologize for these errors. To the best of our knowledge, everything in the corrected article is accurate as presented.

The article has been corrected accordingly.

